# Circular RNA Expression Alteration and Bioinformatics Analysis in Rats After Traumatic Spinal Cord Injury

**DOI:** 10.3389/fnmol.2018.00497

**Published:** 2019-01-14

**Authors:** Chuan Qin, Chang-Bin Liu, De-Gang Yang, Feng Gao, Xin Zhang, Chao Zhang, Liang-Jie Du, Ming-Liang Yang, Jian-Jun Li

**Affiliations:** ^1^School of Rehabilitation Medicine, Capital Medical University, Beijing, China; ^2^China Rehabilitation Science Institute, Beijing, China; ^3^Center of Neural Injury and Repair, Beijing Institute for Brain Disorders, Beijing, China; ^4^Department of Spinal and Neural Functional Reconstruction, China Rehabilitation Research Center, Beijing, China; ^5^Beijing Key Laboratory of Neural Injury and Rehabilitation, Beijing, China

**Keywords:** circular RNA, spinal cord injury, microarray, bioinformatics, rats

## Abstract

Spinal cord injury (SCI) is mostly caused by trauma. As primary mechanical injury is unavoidable in SCI, a focus on the pathophysiology and underlying molecular mechanisms of SCI-induced secondary injury is necessary to develop promising treatments for SCI patients. Circular RNAs (circRNAs) are associated with various diseases. Nevertheless, studies to date have not yet determined the functional roles of circRNAs in traumatic SCI. We examined circRNA expression profiles in the contused spinal cords of rats using microarray and quantitative reverse transcription-PCR (qRT-PCR) then predict their potential roles in post-SCI pathophysiology with bioinformatics. We found a total of 1676 differentially expressed circRNAs (fold change ≥ 2.0; *P* < 0.05) in spinal cord 3 days after contusion using circRNA microarray; 1261 circRNAs were significantly downregulated, whereas the remaining 415 were significantly upregulated. Then, five selected circRNAs, namely, rno_circRNA_005342, rno_circRNA_015513, rno_circRNA_002948, rno_circRNA_006096, and rno_circRNA_013017 were all significantly downregulated in the SCI group after verification by qRT-PCR, demonstrating a similar expression pattern in both microarray and PCR data. The next section of the study was concerned with the prediction of circRNA/miRNA/mRNA interactions using bioinformatics analysis. In the final part of the study, Gene Ontology (GO) and Kyoto Encyclopedia of Genes and Genomes analyses indicated carbohydrate metabolic process was one of the most significant enrichments and meaningful terms after GO analysis, and the top two signaling pathways affected by the circRNAs-miRNAs axes were the AMP-activated protein kinase signaling pathway and the peroxisome related pathway. In summary, this study showed an altered circRNA expression pattern that may be involved in physiological and pathological processes in rats after traumatic SCI, providing deep insights into numerous possibilities for SCI treatment targets by regulating circRNAs.

## Introduction

Spinal cord injury is a growing public health concern worldwide that is accompanied by permanent neurological impairment and attendant social and economic losses (Yang et al., 2014; Manohar et al., 2017). SCI is mostly caused by trauma (Fan et al., 2017), which involves primary mechanical injury due to rapid direct compression and contusion on the cord and secondary injury, such as hemorrhage, edema, ischemia, cell death and oxidative stress, during the following hours (Zhu et al., 2017b). It is well-known that primary mechanical injury is hardly avoidable. As a result, concentrating on the pathophysiology and underlying molecular mechanisms of SCI-induced secondary injury is necessary to develop promising diagnosis and treatment approaches for SCI patients (Liu et al., 2017a). However, no neuroprotective and regenerative therapies are currently available that can directly produce beneficial effects. Although past decades have seen the rapid development of stem cell transplantation (Nagoshi and Okano, 2017), surgical decompression (Yang et al., 2013; Hu et al., 2015) and high-dose methylprednisolone (Hextrum and Bennett, 2018) in the field of SCI, a multitude of controversies on the efficacy of these approaches remains.

Recent advances in ncRNAs, including lncRNA and miRNA, may be at the point of breaking this impasse. Increasing studies in this field have examined an outstanding outcome that neurologic damage can be alleviated by normalizing the expression levels of certain lncRNAs and miRNAs in rats after SCI (Hwang et al., 2016; Lv, 2017; Zhu et al., 2017a; Zhou et al., 2018a). Circular RNAs (circRNAs) are a special type of endogenous non-coding RNAs formed by back-splicing events via protein-coding exons (Qu et al., 2015). Notably, they have been attracting a wealth of interest because growing research has found that the altered expression of specific circRNAs was closely related to human diseases, such as cancer (Zhou et al., 2018b), neurological diseases (Bai et al., 2018), and cardiac diseases (Salgado-Somoza et al., 2017). In recent years, a selection of studies stated that circRNA expression profiles were significantly altered after traumatic brain injury in mice and rats (Xie et al., 2018; Zhao et al., 2018). Nevertheless, there are no data concerning circRNAs in traumatic SCI so far, and their molecular and intermolecular interactions and key signaling pathways remain to be elucidated. Although the physiological functions of circRNAs are largely unknown, they are expected to regulate the transcription of parent genes, promote rolling circle translation, and help form alternatively spliced mRNAs and sponge miRNAs, namely, ceRNAs (Memczak et al., 2013; Liu et al., 2017b).

We used high-throughput microarray analysis to screen circRNAs expression patterns in the spinal cord of adult rats after traumatic SCI to determine whether the expression levels of circRNAs were altered and lay a foundation for future work. Subsequently, we selected five differentially expressed circRNAs for low-throughput validation by qRT-PCR. Then, we used bioinformatics tools to predict their putative biological functions with miRNA and mRNA by establishing circRNA/miRNA/mRNA networks and to identify feasible functions of all mRNAs regulated by these specific circRNAs in traumatic SCI.

The purpose of this investigation was to explore the circRNA expression profiles in rats after traumatic SCI, to determine the potential roles of these differentially expressed circRNAs in post-SCI pathophysiology processes.

## Materials and Methods

### Animals and Experimental Groups

This study was carried out in accordance with the principles of the Basel Declaration and recommendations of the National Institute of Health Guide for the Care and Use of Laboratory Animals (NIH Publications No. 8023, revised 1978). The protocol was approved by the Institutional Animal Care and Use Committee of Capital Medical University. Female Sprague–Dawley rats (age: 10–12 weeks; weight: 250–300 g) were purchased from the Animal Care Center of Academy of Military Medical Sciences (Beijing, China). Rats were housed in a temperature-controlled (20–28°C) and light-controlled (12-h light/dark cycle) room. They were habituated to the housing conditions for at least 7 days before SCI. Additionally, the animals had free access to standard rat chow and tap water; however, food was withheld overnight before surgery.

Twelve rats were randomly assigned to two groups using a computer-generated randomization schedule: rats in the sham control group (*n* = 6) were treated with laminectomy alone without contusion, and rats in the SCI group (*n* = 6) were subjected to laminectomy plus contusion. The operators performing the surgeries were blinded to the experimental groups. Before and after surgery, our two experienced researchers performed a behavioral test of rats in the two groups using the BBB (Basso, Beattie, and Bresnahan) score, which is frequently used to observe the locomotor function of rats (Scheff et al., 2002). The BBB score ranges from 0 (no hindlimb movement) to 21 (normal movement), and the final score was given by consensus.

### Contusion SCI Model and Tissue Collection

In this study, the contusion injury method was adopted to trigger moderate injuries in rat models. Briefly, all rats were anesthetized by intraperitoneal injection of 0.4 mg/g body weight 10% chloral hydrate (Kermel, Tianjing, China), and their backs were shaved and sterilized. After suspending the rats in a stereotaxic frame, a 4-cm-long longitudinal midline incision was made to expose the T9–T11 spinal column. Following stripping of the paraspinal muscles, laminectomy was performed at the T10 level to expose the spinal dura mater without tearing it in a sterile condition. Subsequently, rats were clamped by their spinous processes at T9 and T11 with sterilizing forceps, which was followed by spinal cord contusions induced by an Infinite Horizon Impactor (IH-0400 Impactor, Precision Systems and Instrumentation, LLC, United States), leading to a moderate injury at the T10 level. For parameter setting, a standard rat tip impactor size (2.5 mm in diameter), programmable dwell time (1 s) and programmable force levels (225 kDynes) were applied to induce moderate intensity injuries with the NYU weight-drop device (2.5 mm in diameter, 10 g rod, height of 12.5 mm) according to the conversion equation (Basso et al., 1996; Khuyagbaatar et al., 2015). Errors greater than 3% in terms of force levels and fracture of spinal dura mater were not accepted.

After inducing contusions, the operative region was washed gently with warm 0.9% normal saline twice (37°C, 2 mL) to avoid local infection. The cord surface showed signs of subarachnoid hematoma and an intense dark brown/purple color. The rats with swinging tails that quickly retracted their lower limbs immediately after SCI were regarded as eligible as described previously (Hu et al., 2015). Finally, the wound was then sutured in layers. A warm environment was established to maintain body temperature during surgery. Notably, 4 mL of Ringer lactate solution was administered intraperitoneally to supplement electrolytes and body fluids. Rats were then housed in individual cages. Animals were fed with free access to food and water. Penicillin (40000 U, intramuscular injection) was administered daily for 3 days to prevent systemic infection. The bladders were emptied manually every 8 h until the rats were killed.

At 3 days post-surgery, rats in all groups were euthanized with an overdose of 10% chloral hydrate (10 mL/kg), and a 1-cm long segment of spinal cord, including the injury epicenter, was quickly dissected and collected without transcardial perfusion in advance and was then fresh frozen in liquid nitrogen to prevent RNA degradation similarly to a previously reported method (Pang et al., 2016).

### RNA Extraction and Quality Control

Briefly, total RNA was isolated with TRIzol reagent (Invitrogen, Carlsbad, CA, United States) based on the manufacturer’s instructions. After identifying the purity and concentration of total RNA from each sample with a NanoDrop ND-1000 (NanoDrop, Wilmington, DE, United States), the integrity of RNA and gDNA contamination was tested using denaturing agarose gel electrophoresis.

### CircRNA Microarray

Cord tissue samples from rats in the SCI (*n* = 3) and sham groups (*n* = 3) were used in the microarray experiment. As shown in Supplementary Figure 1A, the sample preparations, sample labeling, and microarray hybridizations were performed based on the manufacturer’s standard protocols (Arraystar). First, total RNAs were digested with Rnase R (Epicentre, Inc.) to remove linear RNAs and enrich circRNAs. Second, the enriched circRNAs were amplified and transcribed into fluorescent cRNA using a random priming method (Arraystar Super RNA Labeling Kit; Arraystar). Third, the labeled cRNAs were purified by a RNeasy Mini Kit according to the manufacturer’s instructions (Qiagen). Then, the concentration and specific activity of the labeled cRNAs (pmol Cy3/μg cRNA) were accessed by a NanoDrop ND-1000. Afterward, 1 μg of each labeled cRNA was fragmented by adding 5 μL of 10× blocking agent and 1 μL of 25× fragmentation buffer, and then, the mixture was heated at 60°C for 30 min; finally, 25 μL of 2× hybridization buffer was added to dilute the labeled cRNA. Approximately 50 μL of hybridization solution was dispensed into the gasket slide and assembled onto the circRNA expression microarray slide. Lastly, the slides were incubated for 17 h at 65°C in an Agilent Hybridization Oven. The hybridized arrays were washed, fixed and scanned with an Agilent Scanner G2505C. The microarray experiments in our study were performed by KangChen Bio-tech, Shanghai, China. This standard procedure was also used in other publications (Xie et al., 2018).

### CircRNA Data Analysis

Acquired array images were analyzed using Agilent Feature Extraction software (version 11.0.1.1). As shown in Supplementary Figure 1B, quantile normalization and subsequent data processing were performed with the R software limma package. After quantile normalization of the raw data, low intensity filtering was performed. When comparing two groups for profile differences (SCI versus sham control), the “fold change” (i.e., the ratio of the group averages) between the groups of each circRNA was computed. The statistical significance of the difference was conveniently estimated by a *t*-test. False discovery rate was calculated from Benjamini Hochberg FDR to correct the *P*.

Differentially expressed circRNAs with statistical significance (fold changes ≥ 2.0, *P* < 0.05) between the groups were identified through volcano plot filtering or fold change filtering. Hierarchical clustering was used to show distinguishable circRNAs expression patterns among tissue samples using MeV (Multiple Experiment Viewer^1^). Additionally, the distribution of differentially expressed circRNAs in rat chromosomes and the bar diagram of circRNA categories were performed by GraphPad prism 6.

Next, we first predicted the potential sponging miRNAs for each differentially expressed circRNA with Arraystar’s homemade miRNA target prediction software according to TargetScan (Enright et al., 2003) and miRanda (Pasquinelli, 2012) in order to investigate the functions of dysregulated circRNAs. We ranked miRNA candidates based on the mirSVR and listed the five highest-ranking miRNA candidates for each circRNA.

### qRT-PCR Assay

The expression of five circRNAs randomly selected from microarray analysis was validated using a qRT-PCR assay. Cord tissue samples from rats in the SCI (*n* = 6) and sham groups (*n* = 6) were used in the PCR experiment. As previously reported (Xie et al., 2018), total RNA was isolated using TRIzol reagent according to the manufacturer’s standard protocols (Invitrogen). RNA quantification and quality were accessed by a NanoDrop ND-1000, and RNA integrity was identified by electrophoresis on a denaturing agarose gel. Next, we synthesized cDNA in line with the manufacturer’s instructions. qRT-PCR was performed in a ViiA 7 Real-time PCR System (Applied Biosystems) with PCR master mix (2×, Arraystar). The parameter settings were 95°C denaturation (10 min), 95°C (10 s), and 60°C (60 s), which was repeated for 40 cycles. After the amplification reaction was finished, the procedure was performed as follows: 95°C (10 s), 60°C (60 s), and 95°C (15 s). Glyceraldehyde 3-phosphate dehydrogenase (GAPDH) acted as an internal control to normalize the data. The relative expression levels of circRNAs were calculated using the relative standard curve method (Larionov et al., 2005).

### Prediction for CircRNA/miRNA/mRNA Interactions

The ceRNA hypothesis is that RNA transcripts can crosstalk by competing for common miRNAs, where MREs are the foundation of this interaction (Salmena et al., 2011). Any RNA transcript with MREs might act as a ceRNA, and ceRNAs include pseudogene transcripts, circRNAs and mRNAs; these transcripts can compete for the same MER to mutually regulate it. We constructed the ceRNA network by merging common targeted miRNAs. The circRNA/miRNA/mRNA interactions were predicted in combination with in-house miRNA target prediction software according to TargetScan and miRanda software (Friedman et al., 2009; Huang et al., 2016) to identify the potential targets of miRNAs. Three conditions must exist for the ceRNA network to occur (Salmena et al., 2011). First, the relative concentration of ceRNAs and their miRNAs is clearly important; second, the effectiveness of a ceRNA depends on the number of miRNAs that it can “sponge”; third, not all of the MREs on ceRNAs are equal. Therefore, we accept only the ceRNA-pair relations that passed filtering measures (*P* < 0.05). A graph of the circRNA/miRNA/mRNA network was made using Cytoscape software (version 3.5.1) to visualize these relationships.

Next, we used Gene Ontology (GO^2^) to reveal the biological process, cellular component and molecular function of the target mRNAs. Significant pathways were identified using pathways in the Kyoto Encyclopedia of Genes and Genomes database (KEGG^3^). A *P* < 0.05 indicates the significance of GO and KEGG pathway terms. The FDR was calculated to correct the *P*.

### Statistical Analysis

Statistical analysis was performed using SPSS software (version 21.0, Chicago, IL, United States). The results were shown as the mean ± SEM. Student’s *t*-test was used to compare significant differences between the two groups. A *P* < 0.05 was considered to be statistically significant.

### Database and Accession Numbers

CircRNA microarray data in our study were deposited at the NCBI Gene Expression Omnibus (GEO) under the Accession No. GSE114426.

## Results

### Expression Pattern of CircRNAs in Spinal Cord Tissues After SCI

Prior to injury, the BBB scores of all rats were 21. Rats in the sham group remained at 21, indicating the integrity of the spinal cord during the experimental period. However, in the SCI group, no hindlimb motor performance was observed immediately after anesthesia recovery, which indicates a loss of locomotive function due to the contusion (data not shown).

**Figure 1:**
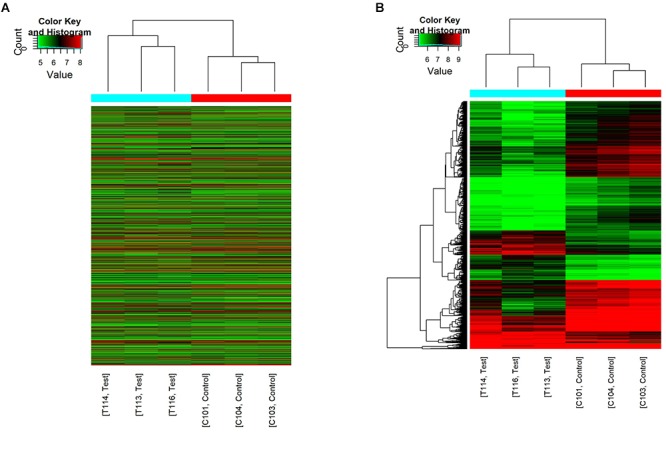
Hierarchical clustering of circRNA expression. C101/103/104 refers to the cord samples from the sham control group, and T113/114/116 refers to the cord samples from the SCI group. **(A)** Hierarchical clustering analysis included all 13279 circRNAs between the sham control and the SCI groups. **(B)** Hierarchical clustering analysis included differentially expressed circRNAs (fold change ≥ 2; *P* < 0.05) between the sham control and the SCI groups.

**Figure 2:**
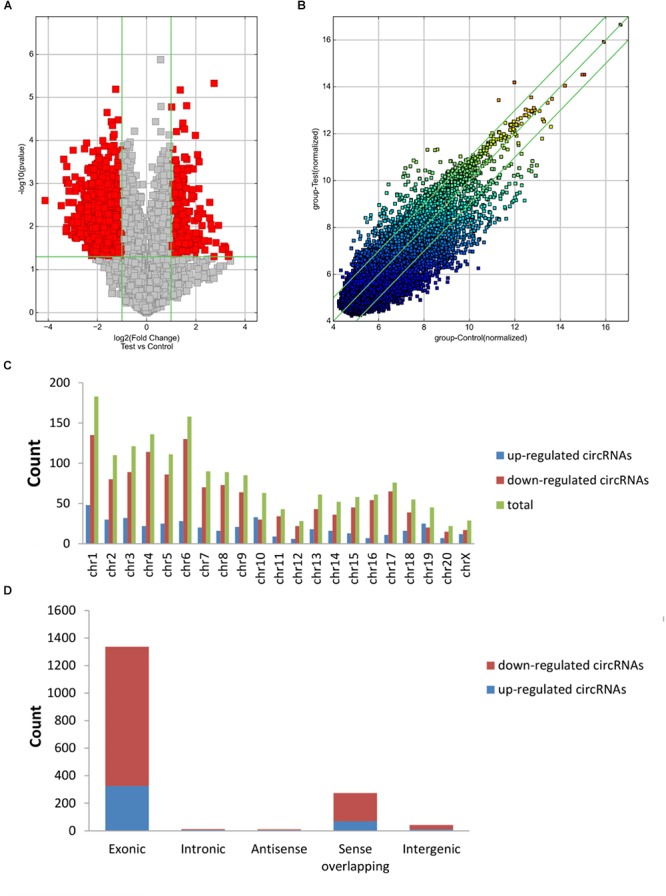
Differences in the circRNA expression profiles between the two groups. **(A)** The scatter plot showed the differences in circRNA expression between the SCI and sham groups. The values of the X and Y axes in the scatter plot are the normalized signal values of the samples (log2 scaled) or the averaged normalized signal values of groups of samples (log2 scaled). The green lines are fold change lines. The circRNAs above the top green line and below the bottom green line indicated more than twofold changes of circRNAs between the two compared samples. **(B)** Volcano plots show the differentially expressed circRNAs with statistical significance (fold change ≥ 2; *P* < 0.05). The vertical lines correspond to 2.0-fold up and down, respectively, and the horizontal line represents a *P* of 0.05; the red point in the plot represents differentially expressed circRNAs with statistical significance. **(C)** The distribution of differentially expressed circRNAs in chromosomes is presented, showing that the dysregulated circRNAs stem from every chromosome. **(D)** The bar diagram of circRNA categories based on gene sources is shown, revealing that most of the circRNAs altered after SCI are exonic.

The first set of questions aimed to identify circRNAs expression levels in the two groups, so a high-throughput microarray assay was used. It can be seen from the box plot (Supplementary Figure 2A) that the normalized intensity values in all samples were not significantly different, suggesting a similar distribution of circRNAs expression profiles in each group. Supplementary Figure 2B showed RNA integrity and the gDNA contamination test. After microarray scanning and normalization, the dysregulated expression of all 13279 circRNAs between groups was detected. A total of 1676 circRNAs were differentially expressed (fold change ≥ 2; *P* < 0.05) between the groups, 1261 of which were significantly downregulated and 415 of which were significantly upregulated in the SCI group. Then, Table 1 provides experimental data on the top 10 upregulated and downregulated circRNAs ranked by fold changes after microarray analysis. Two graphs of hierarchical clustering of circRNAs expression were performed (shown in Figure 1) to visualize these data. Next, volcano plots were constructed using fold change values and P values to visualize differential expression between the two different conditions (Figure 2A), and scatter plots presented the circRNA expression variation (or reproducibility) between the SCI and sham control groups (Figure 2B). Additionally, the distribution of differentially expressed circRNAs in chromosomes is presented in Figure 2C. What stands out in Figure 2D is that the bar diagram of the circRNA category based on gene sources revealed 325 exonic, 6 antisense, 7 intronic, 9 intergenic, and 68 sense overlapping circRNAs that were upregulated. In contrast, 1012 exonic, 4 antisense, 5 intronic, 33 intergenic, and 207 sense overlapping circRNAs were downregulated.

**Table 1:** The top 10 up-regulated and down-regulated circRNAs ranked by fold changes after SCI.

CircRNA	FC	*P*-value	FDR	Chrom	Strand	CircRNA type	Best transcript	GeneSymbol	Regulation
circRNA_005554	17.4927581	0.00249	0.07049	chr15	-	Exonic	NM_031783	Nefl	Down
circRNA_011688	11.0787102	0.00326	0.07374	chr4	+	Exonic	NM_001047116	Rundc3b	Down
circRNA_001668	10.3034657	0.00027	0.05619	chr1	-	Exonic	NM_012506	Atp1a3	Down
circRNA_015152	9.6814247	0.01177	0.09248	chr7	+	Exonic	NM_001271371	Anks1b	Down
circRNA_003787	9.6707443	0.01306	0.09515	chr12	+	Sense overlapping	NM_001105937	Sgsm1	Down
circRNA_005536	9.6595211	0.00082	0.06066	chr15	-	Exonic	XM_003751491	LOC691889	Down
circRNA_013612	9.5445837	0.00081	0.06066	chr6	-	Sense overlapping	NM_078620	Slc8a3	Down
circRNA_013610	9.4741174	0.00040	0.05971	chr6	-	Exonic	NM_078620	Slc8a3	Down
circRNA_015151	9.4653558	0.01049	0.09061	chr7	+	Sense overlapping	NM_001271371	Anks1b	Down
circRNA_009608	9.316022	0.00098	0.06066	chr20	-	Sense overlapping	NM_013189	Gnaz	Down
circRNA_014299	10.2046519	0.04402	0.15222	chr6	+	Exonic	NM_053888	Myt1l	Up
circRNA_014620	9.9983039	0.04890	0.16031	chr6	-	Exonic	NM_020083	Ralgapa1	Up
circRNA_31436	9.258806	0.03142	0.13033	chr18	+	Sense overlapping	NM_012499	Apc	Up
circRNA_002187	9.0443039	0.03409	0.13447	chr10	-	Exonic	XM_002727722	Dnah9	Up
circRNA_017723	9.0003643	0.02898	0.12586	chrX	-	Exonic	XM_229124	Smarca1	Up
circRNA_008695	8.8206292	0.02831	0.12445	chr2	+	Exonic	NM_001013200	Anp32e	Up
circRNA_002186	8.4481357	0.03361	0.13388	chr10	-	Exonic	XM_002727722	Dnah9	Up
circRNA_23780	7.577899	0.00934	0.08796	chr10	+	Exonic	NM_001013117	Phf12	Up
circRNA_014301	7.0536658	0.02369	0.11557	chr6	+	Exonic	NM_053888	Myt1l	Up
circRNA_005470	6.6801172	0.00001	0.02228	chr15	+	Sense overlapping	NM_031056	Mmp14	Up

Lastly, we predicted the five most likely potential target miRNAs for each differentially expressed circRNA. The five highest-ranking miRNA candidates as binding targets of each circRNA are listed in Table 2 (for the top 10 upregulated and downregulated circRNAs).

**Table 2:** The five highest-ranking miRNA candidates for top 10 up-regulated and down-regulated circRNAs.

CircRNA	Predicted miRNA response elements (MREs)				
	MRE1	MRE2	MRE3	MRE4	MRE5
circRNA_005554	rno-miR-547-3p	rno-miR-216a-5p	rno-miR-141-5p	rno-miR-615	rno-miR-328a-3p
circRNA_011688	rno-miR-26b-3p	rno-miR-667-5p	rno-miR-133c	rno-miR-540-3p	rno-miR-18a-5p
circRNA_001668	rno-miR-494-5p	rno-miR-410-5p	rno-miR-205	rno-miR-496-5p	rno-miR-377-5p
circRNA_015152	rno-miR-22-5p	rno-miR-485-5p	rno-miR-488-3p	rno-miR-431	rno-miR-187-3p
circRNA_003787	rno-miR-370-5p	rno-miR-151-5p	rno-miR-346	rno-miR-485-5p	rno-miR-1843b-5p
circRNA_005536	rno-miR-23b-3p	rno-miR-23a-3p	rno-miR-495	rno-miR-493-3p	rno-miR-7a-1-3p
circRNA_013612	rno-miR-182	rno-miR-107-5p	rno-miR-298-5p	rno-miR-31a-5p	rno-miR-6314
circRNA_013610	rno-miR-182	rno-miR-140-3p	rno-miR-17-2-3p	rno-miR-298-5p	rno-miR-6216
circRNA_015151	rno-miR-466b-5p	rno-miR-363-5p	rno-miR-466b-3p	rno-miR-466d	rno-miR-297
circRNA_009608	rno-miR-3084b-5p	rno-miR-3084c-5p	rno-miR-3558-3p	rno-miR-3541	rno-miR-336-5p
circRNA_014299	rno-miR-673-5p	rno-miR-3575	rno-miR-370-3p	rno-miR-466c-5p	rno-miR-672-5p
circRNA_014620	rno-miR-329-5p	rno-miR-22-5p	rno-miR-3568	rno-miR-760-3p	rno-miR-185-3p
circRNA_31436	rno-miR-466b-3p	rno-miR-466c-3p	rno-miR-466b-4-3p	rno-miR-466b-2-3p	rno-miR-297
circRNA_002187	rno-miR-207	rno-miR-27a-3p	rno-miR-135b-5p	rno-miR-484	rno-miR-27b-3p
circRNA_017723	rno-miR-216b-5p	rno-miR-153-5p	rno-miR-28-5p	rno-miR-130a-5p	rno-miR-329-5p
circRNA_008695	rno-miR-880-5p	rno-let-7g-5p	rno-miR-1306-3p	rno-miR-3084a-3p	rno-miR-3084d
circRNA_002186	rno-miR-6331	rno-miR-207	rno-miR-135b-5p	rno-miR-3583-3p	rno-miR-320-5p
circRNA_23780	rno-miR-204-3p	rno-miR-328a-5p	rno-miR-423-5p	rno-miR-138-5p	rno-miR-3573-5p
circRNA_014301	rno-miR-672-5p	rno-miR-3593-5p	rno-miR-3575	rno-miR-1306-5p	rno-miR-466b-5p

### Verification of qRT-PCR

**Figure 3:**
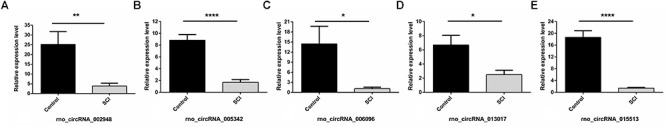
Validation of five selected circRNAs using qRT-PCR. Compared with the sham control, rno_circRNA_005342, rno_circRNA_015513, rno_circRNA_002948, rno_circRNA_006096, and rno_circRNA_013017 in the SCI group were all significantly downregulated after SCI after validation by PCR assay in 12 samples **(A–E).** The data were normalized using the mean ± SEM (*n* = 6 per group). ^∗^*P* < 0.05, ^∗∗^*P* < 0.01, ^∗∗∗∗^*P* < 0.0001.

Overall, 1261 differentially expressed circRNAs were listed after we filtered the high-throughput microarray assay data; therefore, the accuracy of the data needed verification. Next, five circRNAs with relatively high fold changes and similar tissue sample distributions within each group were randomly selected for low-throughput verification with qRT-PCR. Compared with the sham control, rno_circRNA_005342, rno_circRNA_015513, rno_circRNA_002948, rno_circRNA_006096, and rno_circRNA_013017 in the SCI group were all significantly downregulated after SCI, suggesting a similar expression pattern in these candidate circRNAs in both the microarray and PCR data, as shown in Figures 3A–E. Table 3 provides the fold change and *P*-value for each candidate circRNA between groups in terms of microarray and PCR analysis to make data interpretation more straightforward. As presented in Table 4, the sequences of the primers used for qRT-PCR analysis of circRNA are listed. We also provided amplification plots and melt curve plots of these candidate circRNAs and GAPDH in Supplementary Figures 3, 4.

**Table 3:** Comparison for candidate circRNAs expression in microarray and PCR.

CircRNAs	Microarray			PCR		
	FC	*P*-value	Regulation	FC	*P*-value	Regulation
rno_circRNA_002948	5.72	0.0064	Down	6.52	0.0096	Down
rno_circRNA_005342	5.59	0.0023	Down	5.18	<0.0001	Down
rno_circRNA_006096	6.36	0.0048	Down	12.77	0.0296	Down
rno_circRNA_013017	4.54	0.0097	Down	2.68	0.0184	Down
rno_circRNA_015513	5.98	0.0130	Down	13.12	<0.0001	Down

**Table 4:** Sequences of primers used for qRT-PCR assay.

Gene name	Primer sequence	Ta Opt (°C)	Product size (bp)
GAPDH (RAT)	F: 5′-GCTCTCTGCTCCTCCCTGTTCTA-3′ R: 5′-TGGTAACCAGGCGTCCGATA-3′	60	124
rno_circRNA_002948	F: 5′-GGACTTGGAGTCTTCCGATGAG-3′ R: 5′-CAGAAGAAAGCAAAAACCCGTA-3′	60	140
rno_circRNA_005342	F: 5′-CCTCCTCTTCTTCCTTCTTCTG-3′ R: 5′-AGGTACAAAACCACAGTCCTGG-3′	60	110
rno_circRNA_006096	F: 5′-GGAACAGTCTTCAGAAAATGCT-3′ R: 5′-GGGTTGAAGGAAAAGCAGTATA-3′	60	64
rno_circRNA_013017	F: 5′-ATATTTGCTGCTCGTGAATTTA-3′ R: 5′-TGGGAGTTGTGGACCTTGT-3′	60	88
rno_circRNA_015513	F: 5′-GAAGCGGCGATCTAGCATT-3′ R: 5′-TATCTGCCCCTCTATGTGGAT-3′	60	126

### Bioinformatic Predictions of CircRNA/miRNA/mRNA Interactions

**Figure 4:**
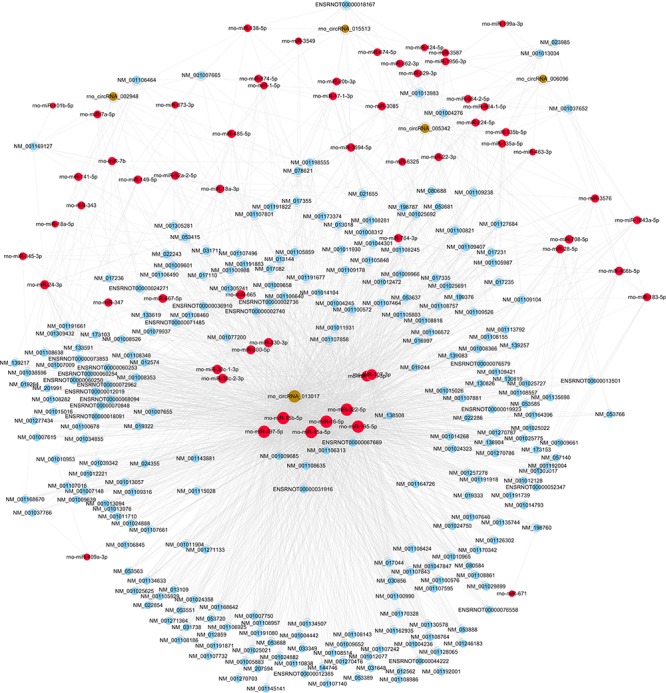
The circRNA/miRNA/mRNA network analysis. The network included the 5 circRNAs, 60 miRNAs, and 253 mRNAs (Nodes with red color are miRNAs; nodes with light-blue color are mRNAs; nodes with brown color are circRNAs).

The five candidate circRNAs validated by qRT-PCR were next selected to construct circRNA/mRNA of miRNA interactions using bioinformatics tools. Notably, an entire network of these interactions was depicted (shown in Figure 4). The results showed that a total of 5 circRNAs, 60 miRNAs and 253 mRNAs were included, presenting a large interaction network. CircRNAs can serve as ceRNA for miRNAs, and miRNAs usually inhibit target mRNAs; therefore, circRNAs may indirectly upregulate target mRNAs by inhibiting the negative regulation of miRNAs.

### GO and Pathway Analysis of Putative Target mRNAs

Based on these interactions, five candidate circRNAs may play an important role in molecular mechanisms by regulating target miRNAs/mRNAs. As a result, we performed GO and pathway analysis of all target mRNAs to provide strong evidence for the further functional verification of these circRNAs.

**Figure 5:**
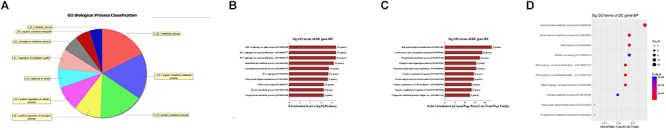
The GO annotations for biological process of target mRNAs regulated by the five candidate circRNAs. **(A)** The pie chart above shows the top 10 counts of the significant enrichment terms. **(B)** The bar plot shows the top 10 enrichment score values of the significant enrichment terms. **(C)** The bar plot shows the top 10-fold enrichment values of the significant enrichment terms. **(D)** The dot plot shows the gene ratio values of the top 10 most significant enrichment terms. Gene ratio: the GOID’s gene ratio value, which equals (Count/List.Total).

**Figure 6:**
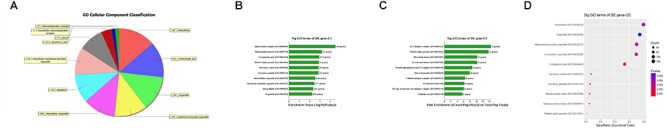
The GO annotations for cellular component of target mRNAs regulated by the five candidate circRNAs. **(A)** The pie chart above shows the top 10 counts of the significant enrichment terms. **(B)** The bar plot shows the top 10 Enrichment Score values of the significant enrichment terms. **(C)** The bar plot shows the top 10-fold enrichment values of the significant enrichment terms. **(D)** The dot plot shows the gene ratio values of the top 10 most significant enrichment terms. Gene ratio: GOID’s gene ratio value, which equals (Count/List.Total).

**Figure 7:**
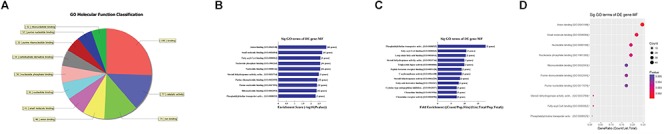
The GO annotations for molecular function of target mRNAs regulated by the five candidate circRNAs. **(A)** The pie chart above shows the top 10 counts of the significant enrichment terms. **(B)** The bar plot shows the top 10 enrichment score values of the significant enrichment terms. **(C)** The bar plot shows the top 10-fold enrichment values of the significant enrichment terms. **(D)** The dot plot shows the gene ratio values of the top 10 most significant enrichment terms. Gene ratio: the GOID’s gene ratio value, which equals (Count/List.Total).

First, GO bioinformatic analysis covers three domains: biological process, cellular component and molecular function. For each part, we showed the following classifications for significantly enriched terms: top 10 counts, top 10-fold enrichment value, top 10 enrichment score value and gene ratio values. The results indicated the following: (1) in terms of biological processes, the most significant enrichment and meaningful terms are metabolic process and RNA splicing, describing a series of biological events (Figures 5A–D). (2) In terms of cellular component, the most significant enrichment and meaningful terms are intracellular parts and complexes, describing the components of a cell (Figures 6A–D). (3) In terms of molecular function, the most significant enrichment and meaningful terms are binding events, suggesting a functional role at the molecular level (Figures 7A–D).

**Figure 8:**
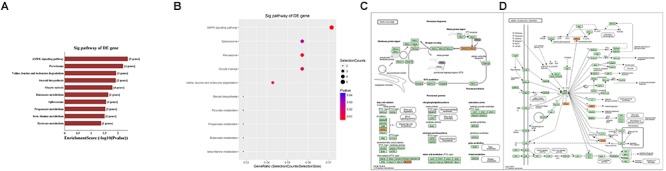
KEGG pathway analysis of target mRNAs regulated by the five candidate circRNAs. **(A)** The bar plot shows the top 10 enrichment score values of the significantly enriched pathway. **(B)** The dot plot shows the gene ratio values of the top 10 most significantly enriched pathways. **(C)** The AMP-activated protein kinase (AMPK) signaling pathway and **(D)** the peroxisome related pathway are shown. Yellow marked nodes are associated with downregulated genes, orange marked nodes are associated with upregulated or only whole dataset genes, and green nodes have no significance.

Second, the top 10 enrichment score value of significantly enriched pathways based on KEGG pathway analysis is presented in Figure 8A. In addition, the dot plot showed the gene ratio value of the top 10 most significantly enriched pathways (Figure 8B). The results suggest that these target genes may play a vital role in multiple metabolic activities, and the top two significant pathways are the AMPK signaling pathway and the peroxisome related pathway (Figures 8C,D).

## Discussion

In the present study, we found that an array of circRNAs were differentially expressed in the early stage after SCI based on circRNA microarray detection. After validation by qRT-PCR, five candidate circRNAs were selected for further prediction. Next, using bioinformatics tools, circRNA/miRNA/mRNA interactions were constructed according to ceRNA mechanisms, indicating a close relationship between circRNAs, miRNAs, and their target mRNAs. Notably, we predicted that the target mRNAs of these confirmed circRNAs may regulate various biological processes, cellular components and molecular functions, suggesting the functional roles of these circRNAs in pathophysiologic processes. Lastly, target mRNAs were revealed to participate in multiple cell signaling pathways of metabolism, including the AMPK signaling pathway and peroxisome activity, which play a pivotal role in the regulation of cell energy homeostasis, significantly affecting cell survival, death, differentiation, proliferation, and inflammation (Chen et al., 2018b; Di Cara et al., 2018). Taken together, the observations from this study suggest that these altered circRNAs are extensively involved in pathophysiologic processes after traumatic SCI. In reviewing the literature, no studies have focused on screening circRNA expression patterns in any model with traumatic SCI. Given this finding, the present study, to the best of our knowledge, provides the first comprehensive assessment of circRNA expression patterns and subsequent functional prediction after traumatic SCI, which offers strong evidence for further investigation.

Most of the human genome is composed of ncRNAs, which are widely involved in physiological and pathological activities and are closely related to many diseases (Dimartino et al., 2018; Sun et al., 2018). miRNAs and lincRNAs, two important types of ncRNAs, have caused interest due to their multiple functions in diverse pathophysiological events (Nicolas, 2017). Prior studies have noted that miRNAs and lincRNAs, as key factors, regulate the molecular mechanisms behind secondary injury after SCI, including inflammation, blood–brain damage, apoptosis, autophagy, oxidative stress, edema, endoplasmic reticulum stress, and demyelination (Shi et al., 2017). Interestingly, normalizing the level of dysregulated miRNAs and lincRNAs can alleviate the pathological changes after SCI (Hu et al., 2013). circRNAs, a recent rising star of ncRNAs, are endogenously expressed as single-stranded and covalently closed circular molecules that are enriched in mammals. Compared to miRNAs and lincRNAs, they are more stable due to their unique covalently closed loop and specific tertiary structures, which offer more possibilities to act as ideal biomarkers or novel therapeutic targets. Recent growing evidence has shown that circRNAs are implicated in various biological processes and human illness, such as cancers, cardiovascular diseases, and neurological disorders (Floris et al., 2017; Chen et al., 2018a; Hu et al., 2018). As an example, in Alzheimer’s disease, Cdr1as is downregulated, thus regulating the downstream target gene and ultimately affecting the progression of disease (Zhao et al., 2016). In terms of trauma to the central nervous system, an report stated for the first time that a total of 192 circRNAs were observed to be differentially expressed (FC ≥ 1.5 and *P* < 0.05) after traumatic brain injury, 98 of which were upregulated and 94 of which were downregulated, indicating the potential roles of these altered circRNAs in pathophysiologic processes after traumatic brain injury^19^. Another study (Zhao et al., 2018) provided evidence concerning circRNA expression alteration in exosomes from the brain extracellular space after traumatic brain injury in mice, which broadens the horizon of research on circRNAs. However, no previous studies have profiled circRNA expression in the spinal cord after SCI. As a result, we put forward a hypothesis that circRNAs expression is significantly altered after traumatic SCI in rats. Finally, after validation, the striking results in our study indicated that circRNAs were altered significantly in rat spinal cord after SCI compared to sham controls.

The relationship between circRNAs and their potential target genes was examined to investigate the functions of these dysregulated circRNAs after screening their expression patterns. Mounting evidence has reported that circRNA can serve as ceRNAs for miRNAs, namely, as miRNA sponges (Liu et al., 2017b; Bai et al., 2018). A circRNA may contain multiple miRNA binding sites and may have adsorptive and suppressive effects on miRNAs. Thus, circRNAs can remove the inhibitory effect of miRNAs on their target mRNAs, indirectly upregulating these target mRNAs. As an example, in the plasma of acute ischemic stroke patients and in a mouse stroke model, circDLGAP4 was significantly downregulated, functioning as an endogenous miR-143 sponge to inhibit miR-143 activity and leading to the inhibition of target gene expression (Bai et al., 2018). In addition, in bladder carcinoma, it has been demonstrated that over-expression of circTCF25 could sponge miR-103a-3p and miR-107, increase CDK6 expression, and promote proliferation and migration *in vitro* and *in vivo* (Zhong et al., 2016). Interestingly, several studies stated that a protein can be translated from circRNAs in human cells driven by N6-methyladenosine22, suggesting a possible translatable function of circRNAs (Du et al., 2017; Yang et al., 2017). However, the number of relevant studies is quite limited, and further studies should be conducted to validate these novel results. As a consequence, based on the function of circRNAs as ceRNAs in regulating the activity of corresponding linear mRNAs by binding miRNAs, we first predicted the potential sponging miRNAs for each differentially expressed circRNA by conserved seed sequence matches. We ranked miRNA candidates based on the mirSVR and listed the five highest-ranking miRNA candidates for each circRNA, including the five candidate circRNAs. Then, target-binding mRNAs for miRNA candidates were calculated and filtered with bioinformatics tools. In our study, a total of 5 circRNAs, 60 miRNAs, and 253 mRNAs were included to construct circRNA/mRNA of miRNA interactions, which presented a large interaction network for bioinformatic analysis. These results together lay the groundwork for future research into the specific circRNA/miRNA/mRNA network, which is beneficial for investigating the role of circRNAs in regulating the expression of target genes. We will next validate the selected interaction.

In addition, GO analysis and KEGG pathway analysis were performed to functionally annotate the predicted target mRNAs of circRNAs-miRNAs axes. First, the goal of the Gene Ontology Consortium is to produce a dynamic, controlled vocabulary that can be applied to all eukaryotes, even as knowledge of gene and protein roles in cells is accumulating and changing (Ashburner et al., 2000); thus, three independent ontologies accessible on the World Wide Web^4^ are being constructed: biological process, molecular function, and cellular component. Briefly, we found that the target mRNAs are involved in multiple biological processes, cellular signaling pathways, protein activities and gene splicing in rat with SCI. As an example, the carbohydrate metabolic process was found to be one of the most significantly enriched and meaningful terms of biological processes after GO analysis. In addition, one previous study confirmed the central adiposity associations to carbohydrate and lipid metabolism in individuals with complete motor SCI (Gorgey et al., 2011). Thus, the results together suggested the functional roles of the circRNA/miRNA/mRNA network in regulating pathophysiology after SCI. Second, we used KEGG pathway analysis to reveal the potential roles of the target mRNAs regulated by circRNAs-miRNAs axes in diverse biological pathways. According to our annotation, the top two signaling pathways affected by the circRNAs-miRNAs axes were the AMPK signaling pathway and the peroxisome related pathway. Previous studies have explored the relationships between these pathways and pathophysiological processes after SCI. As an example, AMPK is a pivotal regulator of energy homeostasis, and after assessing the influence of longstanding and recent SCI on protein abundance of AMPK isoforms in human skeletal muscle, researchers found that physical/neuromuscular activity is an important determinant of isoform abundance of AMPK (Kostovski et al., 2013). Additionally, another report described the pharmacological activation of peroxisome proliferator-activated receptors due to their anti-inflammatory/antioxidant/anti-excitotoxic/pro-energetic profile in SCI (Esposito and Cuzzocrea, 2011). After pathway analysis, we performed pathway diagrams of the AMPK signaling pathway and the peroxisome related pathway (Figures 8A,B), presenting the most significant core downstream pathways of the circRNAs-miRNAs axes directly.

However, several questions remain to be answered. First and most importantly, the reader should bear in mind that this study was unable to validate the circRNA/miRNA/mRNA network predicted by bioinformatics, and our future work is to identify their functions. Next, the relatively small group size for the PCR experiments may limit the interpretation for candidate circRNAs and subsequent bioinformatics. Thus, the enlarged group size is needed to improve the scientific rigor. Then, it was beyond the scope of this study to examine the potential effect of different time points on the possible dynamic changes in circRNA expression patterns in the contused spinal cords of rats with SCI. Last but not least, we strongly recommend that future research should focus on different animal models and types of SCI to study the expression and functions of circRNAs.

Taken together, the current findings of this study were greatly significant in at least two major respects. On one hand, this study explored, for the first time, the significant alteration of circRNA expression profiles after traumatic SCI in rats. On the other hand, these differentially expressed circRNAs were predicted to closely correlate with post-SCI pathophysiology processes based on bioinformatics analysis. Despite its exploratory nature, this study offered deep insights into many possible treatment targets of SCI by regulating circRNAs.

## Data Availability Statement

The raw data supporting the conclusions of this manuscript will be made available by the authors, without undue reservation, to any qualified researcher.

## Author Contributions

CQ, C-BL, and M-LY performed the research. J-JL designed the research study. D-GY and FG contributed essential reagents or tools. CZ and L-JD analyzed the data. CQ wrote the manuscript. All authors read and approved the final manuscript.

## Conflict of Interest Statement

The authors declare that the research was conducted in the absence of any commercial or financial relationships that could be construed as a potential conflict of interest.

## Footnotes

1 https://sourceforge.net/projects/mev-tm4/files/mev-tm4/

2 http://www.geneontology.org

3 www.genome.jp/kegg

4 http://www.geneontology.org

## Abbreviations

AMPK: AMP-activated protein kinase
ceRNAs: competing endogenous RNAs
circRNAs: circular RNAs
FCs: fold changes
FDR: false discovery rate
GAPDH: glyceraldehyde 3-phosphate dehydrogenase
GO: Gene Ontology
KEGG: Kyoto Encyclopedia of Genes and Genomes database
lncRNA: long non-coding RNA
miRNA: microRNA
MREs: miRNA response elements
ncRNAs: non-coding RNAs
qRT-PCR: quantitative reverse transcription-PCR
SCI: spinal cord injury
