# Supplementary material for: Circular RNA Expression Alteration and Bioinformatics Analysis in Rats After Traumatic Spinal Cord Injury
Source: Front Mol Neurosci. 2019 Jan 14;11:497. doi: 10.3389/fnmol.2018.00497 (PMC6339904; doi:10.3389/fnmol.2018.00497)

Supplementary Material

Circular RNA expression alteration and bioinformatics analysis in rats after traumatic spinal cord injury

**Chuan Qin^1,2,5^, Chang-Bin Liu^1,2,5^, De-Gang Yang^1,2,3,5^, Feng Gao^1,2,3,5^, Xin Zhang^1,2,5^, Chao Zhang^1,2,5^, Liang-Jie Du^1,2,5^, Ming-Liang Yang^1,2,3,4,5^, Jian-Jun Li^1,2,3,4,5, *^**

*** Correspondence:** Dr. Jian-Jun Li, [crrclijj@163.com](mailto:crrclijj@163.com)

**Supplementary Fig 1. Experiment work flow.** (A) The microarray experiment procedure is presented, including sample preparations, sample labeling, microarray hybridizations and array scanning. (B) The flowchart of data analysis is presented, including raw data extraction, circRNAs expression profiles, differently expressed circRNAs and annotation off. circRNA/miRNA interactions.


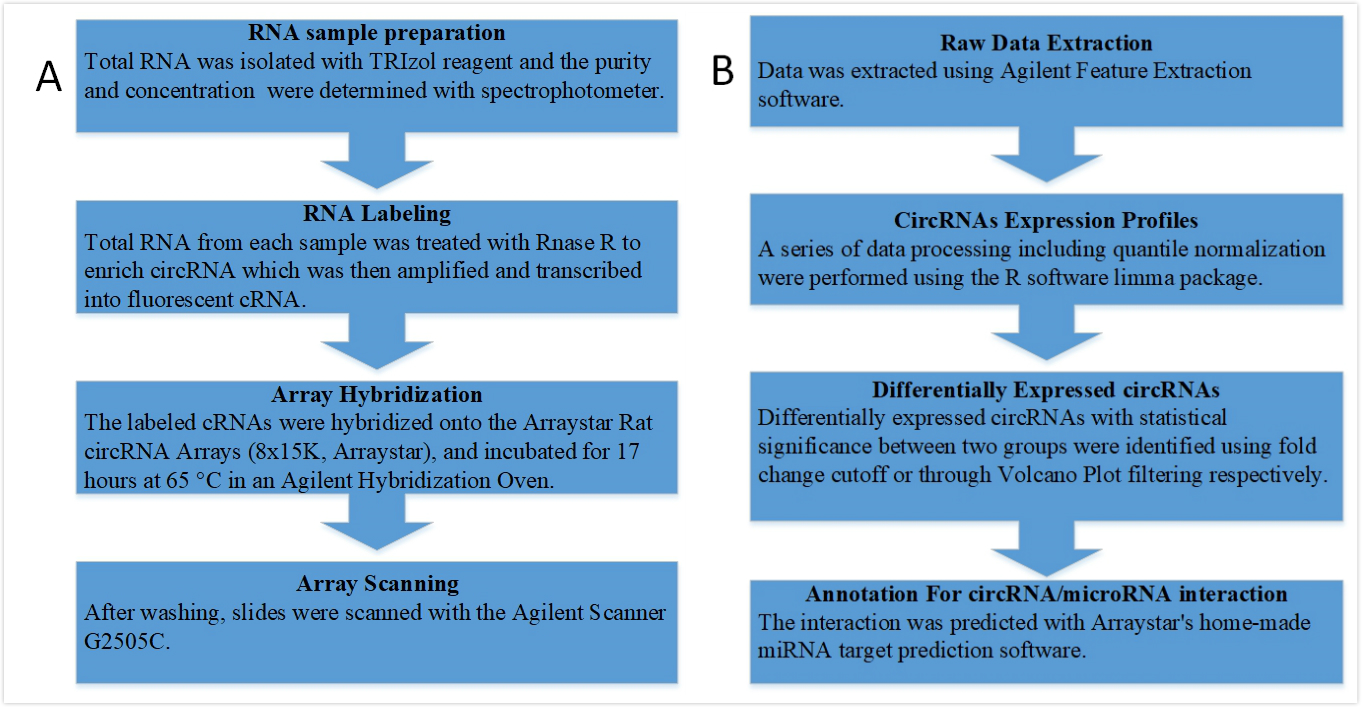


**Supplementary Fig 2.** **Quality control for the experiment.** (A) The box plot showed that the normalized intensity values in all samples were not significantly different, suggesting a similar distribution of circRNAs expression profiles in each group. C101/103/104 refer to the cord samples in the sham control, and T113/114/116 refer to the cord samples in the SCI group. (B) RNA integrity and gDNA contamination test are presented. The 28S and 18S ribosomal RNA bands were sharp and intense. No high molecular weight smear or band migrating above the 28S ribosomal RNA band was detected, suggesting no DNA contamination of the RNA preparation. Lane 1-6: total RNA of sample C103/C104/C101/T116/T114/T113.


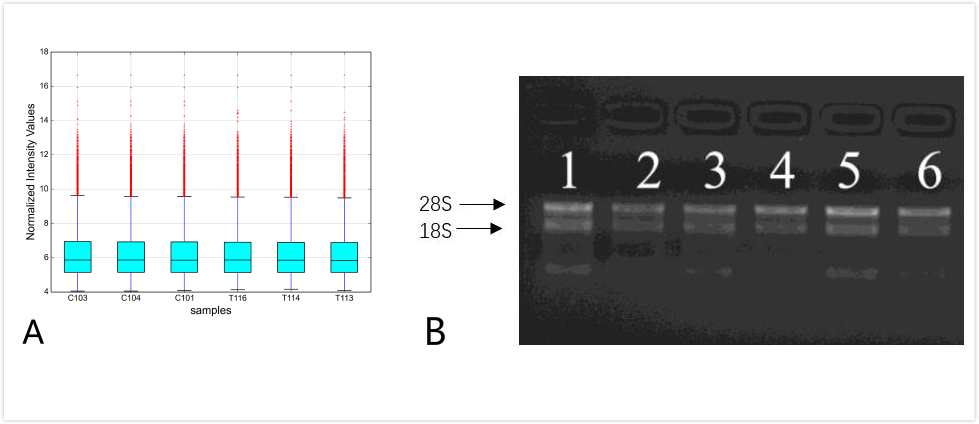


**Supplementary Fig 3. Amplification plots for these candidate circRNAs and GAPDH.** (A\G) GAPDH. (B\H) rno_circRNA_002948. (C\I) rno_circRNA_005342. (D\J) rno_circRNA_006096. (E\K) rno_circRNA_013017. (F\L) rno_circRNA_015513.


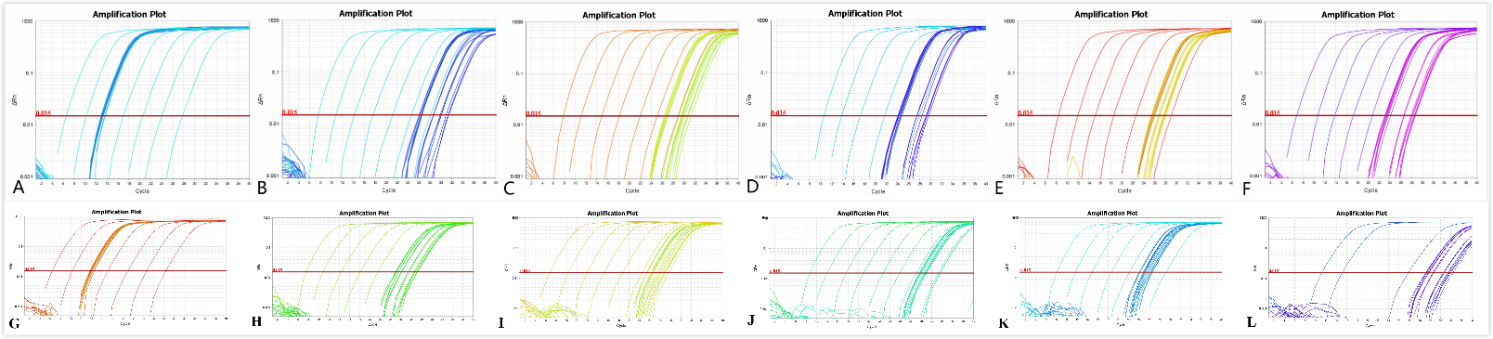


**Supplementary Fig 4.** **Melt curve plots of these candidate circRNAs and GAPDH.** (A\G) GAPDH. (B\H) rno_circRNA_002948. (C\I) rno_circRNA_005342. (D\J) rno_circRNA_006096. (E\K) rno_circRNA_013017. (F\L) rno_circRNA_015513.


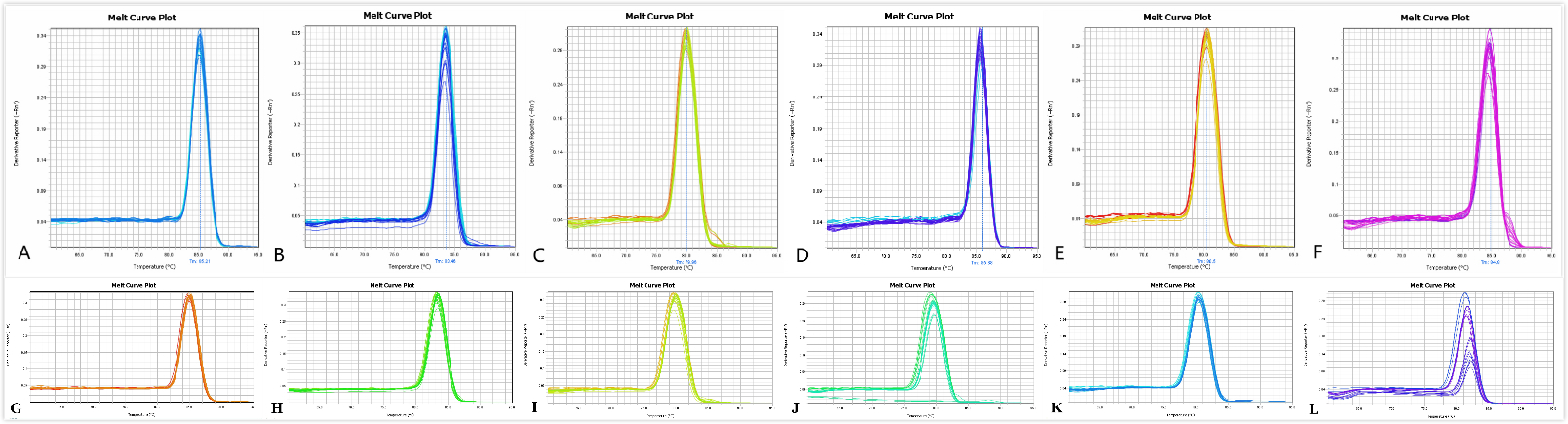

Supplement: Supplementary file 1 [file Data_Sheet_1.docx]
